# Urotensin II receptor determines prognosis of bladder cancer regulating cell motility/invasion

**DOI:** 10.1186/1756-9966-33-48

**Published:** 2014-06-03

**Authors:** Renato Franco, Silvia Zappavigna, Vincenzo Gigantino, Amalia Luce, Monica Cantile, Margherita Cerrone, Gaetano Facchini, Sisto Perdonà, Sandro Pignata, Giuseppe Di Lorenzo, Sergio Chieffi, Giovanni Vitale, Marco De Sio, Alessandro Sgambato, Gerardo Botti, Ali Munaim Yousif, Ettore Novellino, Paolo Grieco, Michele Caraglia

**Affiliations:** 1 Pathology Unit, National Cancer Institute ‘G. Pascale’, Naples, Italy; 2 Department of Biochemistry, Biophysics and General Pathology, Second University of Naples, Naples, Italy; 3 Urogynechologic Oncology Unit, National Cancer Institute ‘G. Pascale’, Naples, Italy; 4 Genitourinary Cancer Section and Rare-Cancer Center, Medical Oncology Division, University Federico II, Naples, Italy; 5 Department of Experimental Medicine, Second University of Naples, Naples, Italy; 6 Department of Clinical Sciences and Community Health, University of Milan and Italian Auxologic Institute IRCCS, Milan, Italy; 7 Urology Unit, Second University of Naples, Naples, Italy; 8 Institute of General Pathology, “Giovanni XXIII” Cancer Research Center, Catholic University of Sacred Heart, Rome, Italy; 9 Department of Pharmacy, University of Naples Federico II, Naples 80131, Italy

**Keywords:** Bladder cancer, Muscle invasive, Non-muscle invasive, Tumor progression, Urotensin-II

## Abstract

**Background:**

Non Muscle Invasive Bladder Transitional Cancer (NMIBC) and Muscle Invasive Bladder Transitional Cancer (MIBC)/invasive have different gene profile and clinical course. NMIBC prognosis is not completely predictable, since the relapse rate is higher than 20%, even in the form of MIBC. The aim of this study is to evaluate if UTR expression can discriminate between NMIBC and MIBC and predict the risk of relapses in NMIBCs.

**Methods:**

We have investigated upon urotensin-II (UII) receptor (UTR) expression *in vivo* in 159 patients affected by NMIBC. The biological role of UTR was also investigated *in vitro*. UTR expression was evaluated in a tissue-micro-array, consisting of normal, NMIBC and invasive bTCC samples.

**Results:**

UTR discriminated between NMIBC and MIBC and showed a significant correlation between low UTR expression and shorter disease free survival in NMIBC. The superagonist UPG84 induced growth suppression at nM concentrations on 3/4 cell lines. Bladder cancer cell treatment with the antagonist urantide or the knock-down of UTR with a specific shRNA significantly blocked both the motility and invasion of bladder cancer cells.

**Conclusions:**

The evaluation of UTR expression can discriminate between NMIBC at high and low risk of relapse. Moreover, our data suggest that UTR is involved in the regulation of motility, invasion and proliferation of bladder cancer cells. High UTR expression is an independent prognostic factor of good prognosis for NMIBC regulating motility and invasion of bladder cancer cells.

## Background

Bladder transitional cell carcinoma (bTCC) represents the 4^th^ most common malignancy in the world [1]. Tumors not invading into *muscularis propria*, include non invasive papillary TCC (pTa), carcinoma in situ (CIS) (pTis), and tumors invading into *lamina propria* (pT1): “Non-Muscle Invasive bladder transitional cancer” (NMIBC). Invasive tumors infiltrate *muscolaris propria:* “Muscle-Invasive bladder transitional cancer” (MIBC) [2,3]. NMIBC tumors have a quite favourable clinical outcome while a high mortality rate has been reported for invasive tumors. About 60% to 70% of NMIBC recur, and about 15% to 25% of patients relapse with invasive bladder cancer [4]. Histopathological stage and grade are currently the two most important factors in determining behaviour and treatment plan for bladder tumors [5]. Prognosis of NMIBC remains unpredictable for both recurrence and progression.

Many biomarkers have been proposed and within them, p53-dependent deregulated pathways seem to be strongly associated to invading tumors [6]. Moreover, bTCC could promote its growth and progression through autocrine/paracrine regulator peptides, such as vascular endothelial growth factor (VEGF) and proepithelin [7-11].

Recently, Urotensin-II receptor (UTR) has been detected in several tumor cell lines but there are conflicting results about its role in tumor progression [12,13].

Treatment with UTII significantly increases human adrenocortical and renal cell carcinoma proliferation [14,15]. In lung adenocarcinoma, it has been demonstrated that treatment with UTII, produced an increased tumor volume *in vitro* and *in vivo*[16].

Our group has recently demonstrated that UTR low expression in prostate adenocarcinoma was significantly associated to both shorter disease free survival (DFS) and overall survival (OS) [12].

In this study, we evaluated UTR expression in a series of bladder cancer cell lines and its involvement in the regulation of biological functions like invasion and motility of bladder cancer cells.

## Methods

### Bladder cancer TMA building

A progressive Tissue Micro-Array (TMA), has been constructed using 130 tissue samples (113 tumour and 17 normal tissues), after pathologic re-evaluation according WHO/ISUP 2007 [17].

Tumor tissues included 36 NMIBC and 77 MIBC. All tumours and controls have been reviewed by two experienced pathologists (RF, GB). Tissue cylinders were brought into one recipient paraffin block using a semiautomated tissue arrayer (Galileo TMA).

The bladder cancer samples were collected from the National Institute of Tumours of Naples after Internal Ethical Institutional approval in compliance with the Helsinki Declaration

### Patients and specimens of prognostic series of NMIBC

159 patients, undergone to bladder biopsy from 2000 to 2008 at the National Cancer Institute “Fondazione Giovanni Pascale” of Naples have been included in this study. 84 (53%) of 159 patients showed relapses. All cases were reviewed according to WHO classification criteria [17].

Medical records have been reviewed for clinical information, including histologic parameters assessed on standard H&E-stained slides.

### Immunohistochemistry analysis

Immunohistochemical staining was done on slides from formalin-fixed, paraffin embedded tissues, to evaluate UTR expression. After protein block, slides were incubated with primary anti-UTR antibody followed by secondary antibody (Novocastra Streptavidin-HRP) and then visualized using a 3,3’-diaminobenzidine. Sections were counterstained with hematoxylin and mounted.

### RNA extraction and analysis

Total RNA was isolated from FFPE biopsies of prognostic series whole section, collected in National Cancer Institute “Fondazione G. Pascale” Institutional Bio-Bank, using High pure FFPE RNA Micro Kit (Roche). RNA was subjected to cDNA synthesis using the Ready To Go You-Primer First-Strand Beads kit (Amersham Biosciences) in a reaction mixture containing random hexamers (Applied Biosystems).

### Real-time PCR

Quantitative RT–PCR was performed in a LightCycler system (Roche) using TaqMan® analysis. All reactions were performed in triplicate. The thermal cycling conditions included a step of 20 sec at 95°C followed by 40 cycles of 95°C for 1 sec and 60°C for 20 sec. Comparative *C*_t_ method was used to determine human *UTR* gene variation, using as reference gene TaqMan Endogenous Controls Human ACTB (β-actin) Endogenous Control (RealTime Designer Assay, Roche). We identified a calibrator cell line (LNCaP) that represents the unitary amount of the target, consequently the samples express *n*-fold mRNA relative to the calibrator.

### Statistical analysis

*UTR* immunohistochemistry expression was evaluated in bladder cancer TMA including normal, NMIBC and invasive samples. The mean and median tissue *UTR* expression, expressed as a percentage of immunoreactive cells, was calculated. Kruskal-Wallis test identified differences in median expression values. Selection of the median value as cut-off score was based on evaluation of the distribution of UTR scores. Differences in the number of negative and positive cases were analyzed using a test of equal proportions.

UTR expression was then evaluated on a prognostic series of NMIBC with complete clinical-pathological information.

Association between UTR expression and other molecular and clinical-pathological parameters was calculated using contingency table methods and tested for significance using the Pearson chi-squared test. Univariate and multivariate relative risks have been calculated using the COX proportional hazards regression. All calculations have been performed using the SPSS (Statistical Package for the Social Science rel.13) software (Chicago, IL) and the results have been considered statistically significant when P-value has been ≤ 0.05.

### Cell lines and cell proliferation by MTT assay

hUII and urantide, the agonist–antagonistic compounds of UII, UPG83, UPG84 and UPG85 were all provided by Prof. P. Grieco [18].

HT1376, MCR, T24 and RT112, cell lines of human bladder cancer, were provided by ATCC. HT1376 and T24 are a grade 3 whereas RT112 is a grade 2 urinary bladder cell line. Cell lines were plated in 96-well plates and one day later were treated with different compounds at concentrations ranging from 10 to 1,000 nM (urantide, UII, UPG83 and UPG85) or with concentrations ranging from 10 to 2,000 nM (UPG84). Cell proliferation was evaluated by MTT assay [19].

### Western blot analysis

Total proteins were prepared as described [19]. Membranes were incubated with the following primary antibodies: (a) anti-UTR; (b) anti- α-tubulin. Bound antibodies were detected by horseradish peroxidase-conjugated secondary antibodies, followed by enhanced chemiluminescence [19].

### FACS analysis of UTR expression in bladder cancer cells

For determination of cell surface UTR expression, analysis was performed using indirect UTR staining at FACS. We have seeded and treated or not cells with 10 nM urantide or UPG84 for 72 h. After treatment, cells were centrifuged and 4% paraformaldehyde was added for 15 min at 4°C in the dark. Cells were incubated in PBS/BSA for 10 min at 4°C and subsequently with a primary rabbit polyclonal antibody raised against human UTR (GPR14) or with an irrelevant immunoglobulin (IgG1) or in PBS and processed as previously described [19].

### Invasion and motility assays

For invasion assays, 8 μm inserts (Falcon) were employed and Matrigel TM (Sigma) was diluted in serum-free medium. Subsequently, assays were performed as previously reported [20].

## Results

### UTR expression was higher in NMIBC

**Figure 1:**
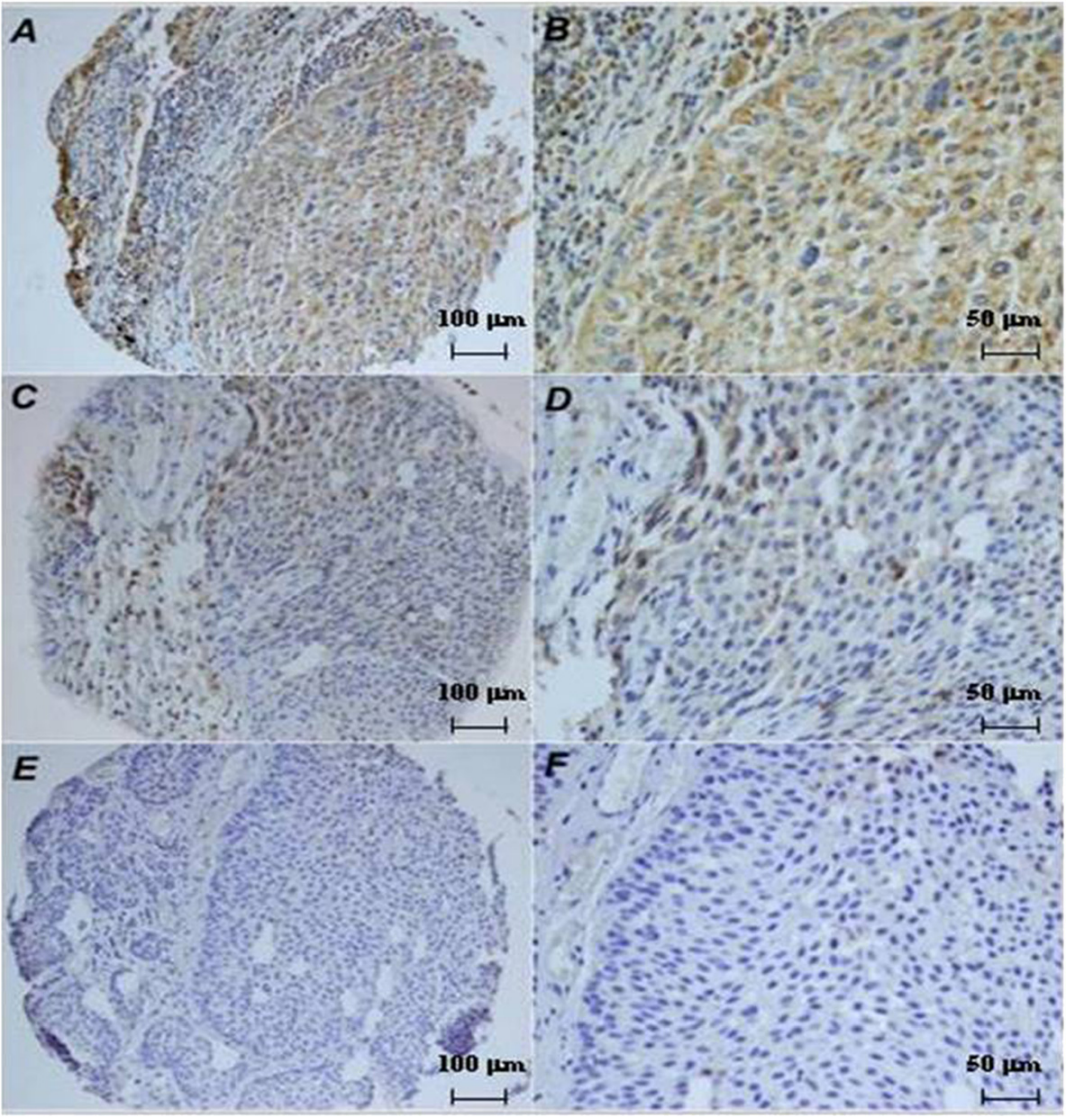
**Immunohistochemical UTR expression in a progressive Bladder TMA. A,B)**: High UTR expression in NMIBC (20x and 40x); **C,D)**: low UTR expression in MIBC (20x and 40x). **E,F)**: negative UTR expression in invasive tumor (20× and 40×).

We have evaluated UTR expression on a progressive bladder TMA. We have found a mean expression of UTR of about 16.67% and 13.57% for NMIBC and MIBC, respectively. Percentage of negative cases was significantly higher in MIBC than NMIBC. Pearson chi-squared test showed significant higher UTR expression in NMIBC (p = 0.0001) (Figure 1). These results suggest a higher expression of UTR in NMIBC.

### UTR expression correlated with low risk of relapses in NMIBCs

Our series included 125 males and 34 females, 118 (74%) older than 60 years of age (mean age 68, range from 40 to 88 years). The anatomic sites of the tumour were lateral wall in 87 cases (55%), cupola in 13 cases (8%), trigon in 51 cases (32%). Relapses have been recorded in 75 patients (47%), from 1 (57) to 6 (1) total episodes (Table 1).

**Table 1:** Clinical-pathological characteristics of the Superficial TCC patients

		**Low UTR expression**	**High UTR expression**
***Patients without relapse (84)***		38	46
**Sex**	Male	28	37
	Female	10	9
**Grade**	High grade	11	8
	Low grade	27	38
**Infiltration**	No	26	35
	Lamina propria	12	11
***Patients with subsequent relapse (75)***		56	19
**Sex**	Male	47	13
	Female	9	6
**Grade**	High grade	16	6
	Low grade	40	13
**Infiltration**	No	34	11
	Lamina propria	22	8

**Figure 2:**
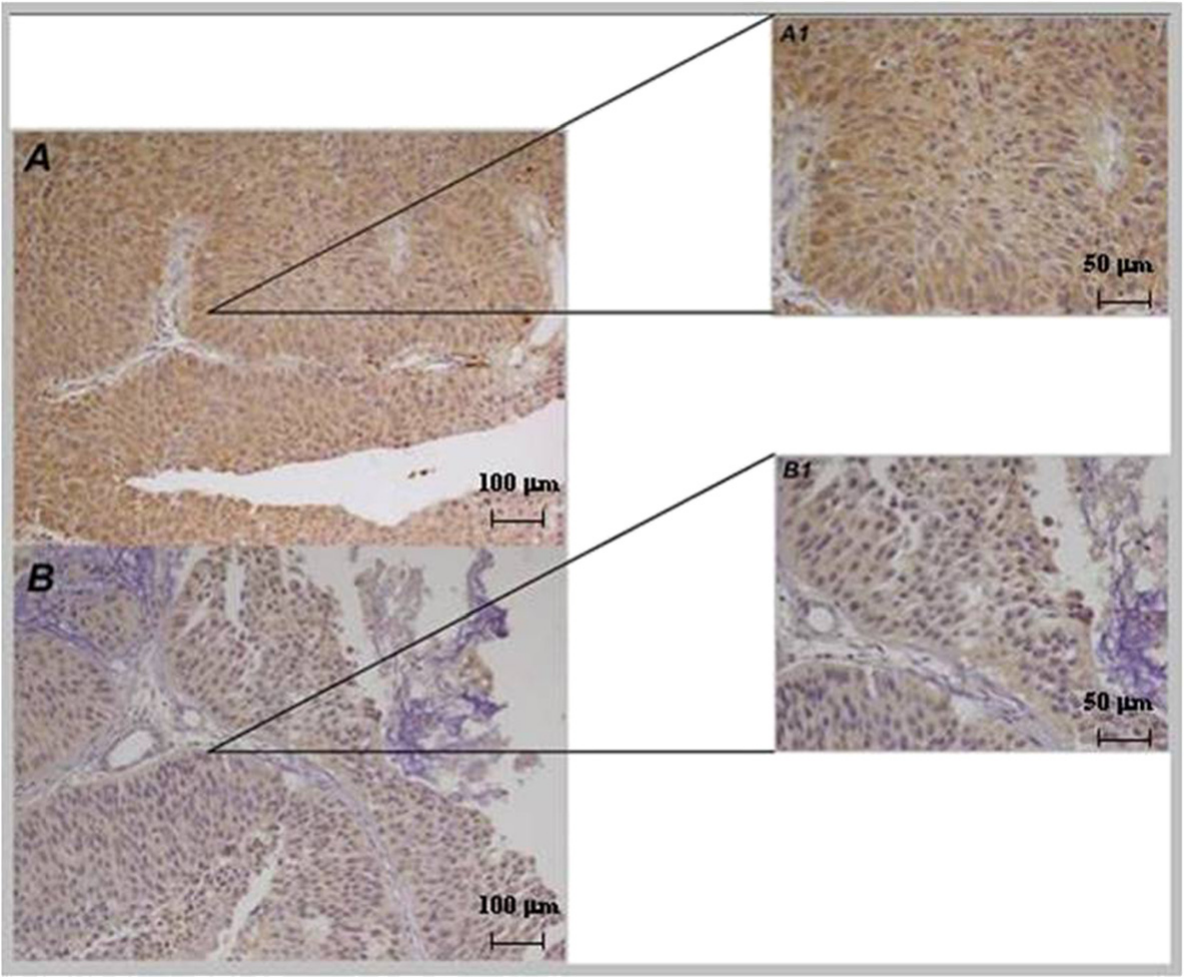
**Immunohistochemical UTR expression in NMIBC. A**, A1): High UTR expression (20x, 40x) in a patient without recurrence; **B**, B1): low UTR expression (20×, 40×) in a patient with recurrence.

**Figure 3:**
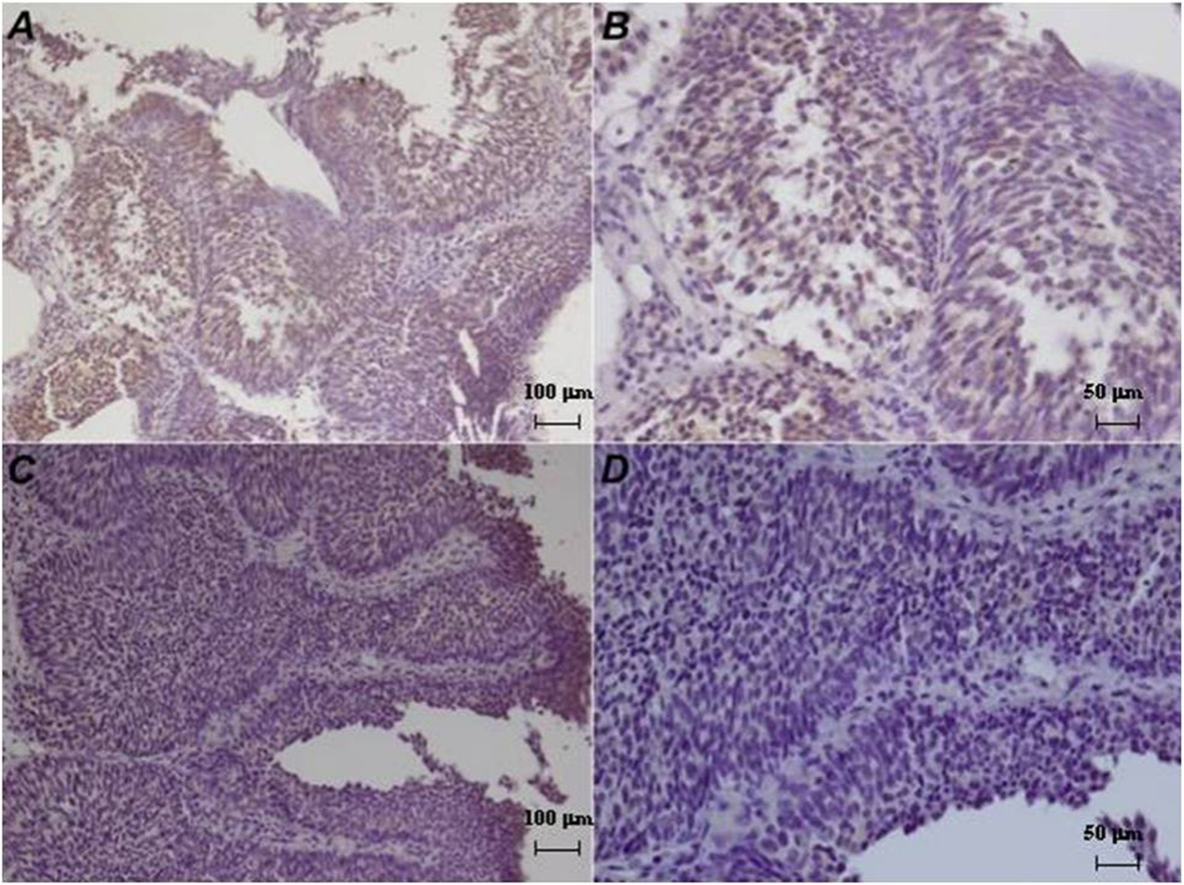
**Immunohistochemical UTR expression in a patient with no recurrence. A**, **B**: High UTR expression (20×, 40×) in a patient who has not developed a relapse; **C**, **D**: low UTR expression (20×, 40×).

High-grade primitive tumours have been observed in 41 patients (26%); the relapse tumor grade was the same of primitive tumors in 71 cases (94%), while 4 cases (6%) of the relapsed tumours showed higher grade than the primary. In 53 cases (33%) *lamina propria* infiltration was observed (Table 1).High expression was recorded when positive cells were > 30%. Patients with low expression of UTR were 94 (59%), in particular 38 (40%) without recurrences and 56 (60%) with histologically documented recurrences. Moreover, 65 patients (41%) showed high expression of UTR, 46 (71%) without recurrences and 19 (29%) with recurrences. High UTR expression was significantly associated to low grade carcinoma (p = 0.044) while low expression was associated to cases that have developed at least one relapse (p = 0.001). Moreover, UTR expression in relapses was significantly lost respect to primary tumors (p = 0.025) (Figures 2 and 3).

The decrease of the correlation between high UTR expression and absence of relapse was likely due to the short time of observation that could explain the presence of some patients who will develop the relapse in the following observation time (likely expressing low UTR levels).

### UTR expression was directly correlated with DFS

**Figure 4:**
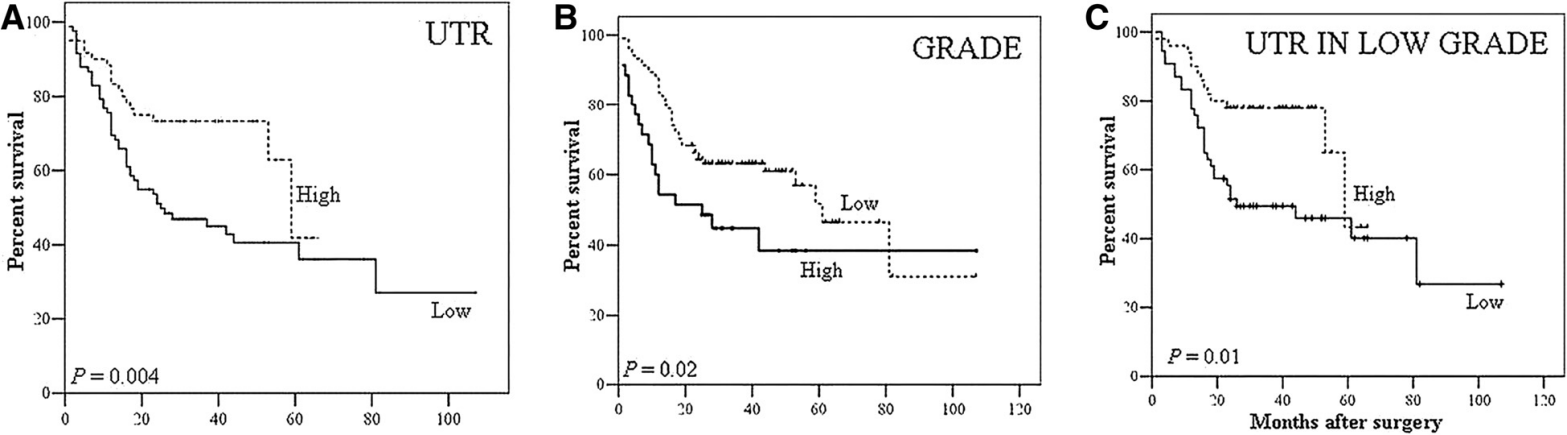
**Kaplan–Meier survival curve. A)** Association between low UTR expression and shorter time of relapse. **B)** Association between tumor grade and shorter time of relapse. **C)** Shorter disease-free survival is associated to low UTR expression also in the low grade carcinoma group.

Considering only first relapse, we recorded a significant association between low UTR expression and shorter time to relapse (p = 0.004). As expected tumor grade was associated to recurrence (p = 0.02) (Figure 4). Also considering low grade carcinoma group, shorter DFS was associated to UTR low expression (p = 0.01) (Figure 4).

In a multivariate analysis including UTR expression and grade, only UTR appeared to be an independent prognostic factor (p = 0.025).

### UTR protein expression modulation was due to changes in its mRNA levels in bTCC

**Figure 5:**
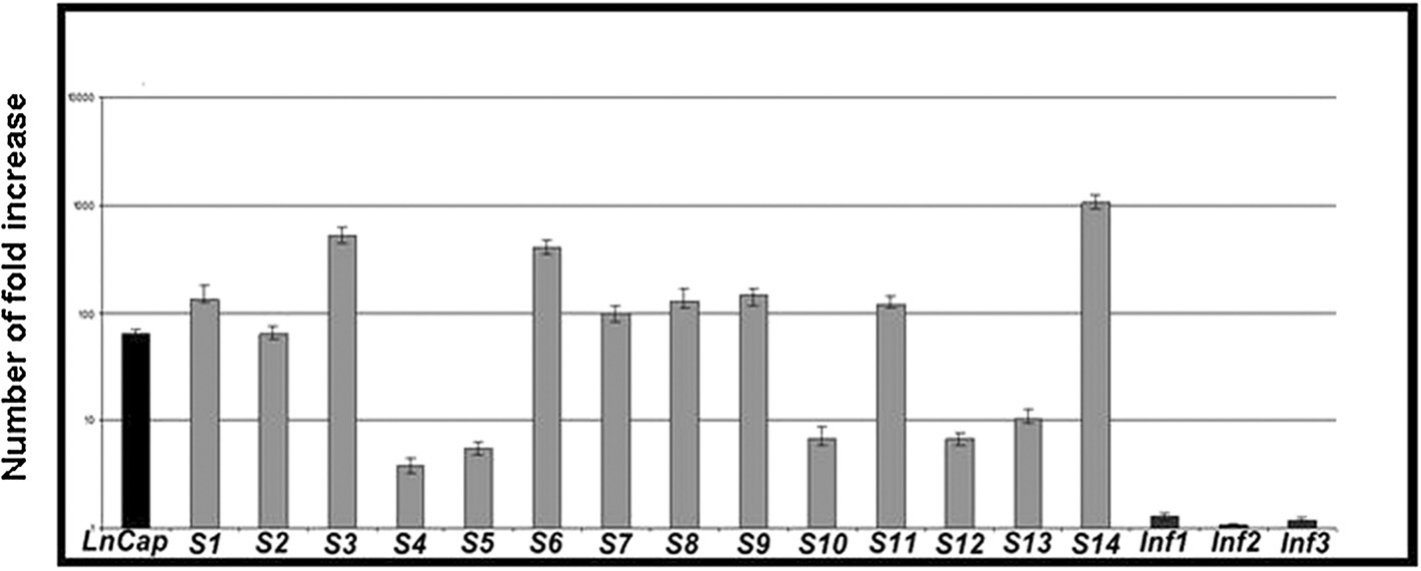
**Real time UTR mRNA quantification in bladder samples.***LNCaP:* calibrator; *S:* superficial bladder tumors; *Inf:* invasive bladder tumor. Data were expressed as mean ± standard deviation (SD, n = 3).

UTR gene expression was evaluated on LNCaP cell line and 14 NMIBC and 3 invasive MIBC samples by Real-Time PCR quantification.In LNCaP, UTR expression was moderate. In 4 NMIBC samples the expression was low while in 3 samples the increase of expression was moderate. The most part of NMIBC showed a significant increase in UTR mRNA expression (Figure 5). UTR gene expression was very low in all invasive bTCC selected (Figure 5). Moreover, UTR mRNA levels correlated with the expression of the protein in all examined cancer samples.

### *In vitro* effects of UII, Urantide, and other agonist/antagonist ligands (UPG85, UPG84 and UPG83) on bladder cancer

**Figure 6:**
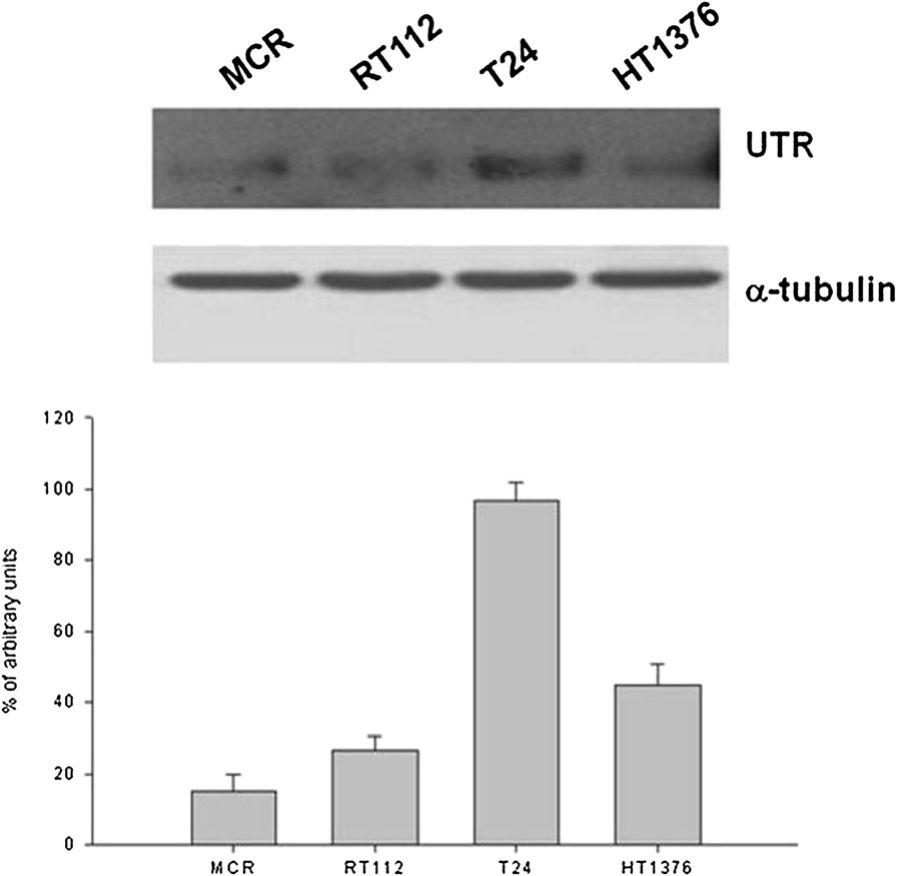
**Expression of UTR by Western blotting.** Cell lines of human bladder cancer are MCR, RT112, T24 and HT1376. The housekeeping protein α-tubulin was used as loading control. Bars, SDs. The experiments were performed at least three different times and the results were always similar.

**Figure 7:**
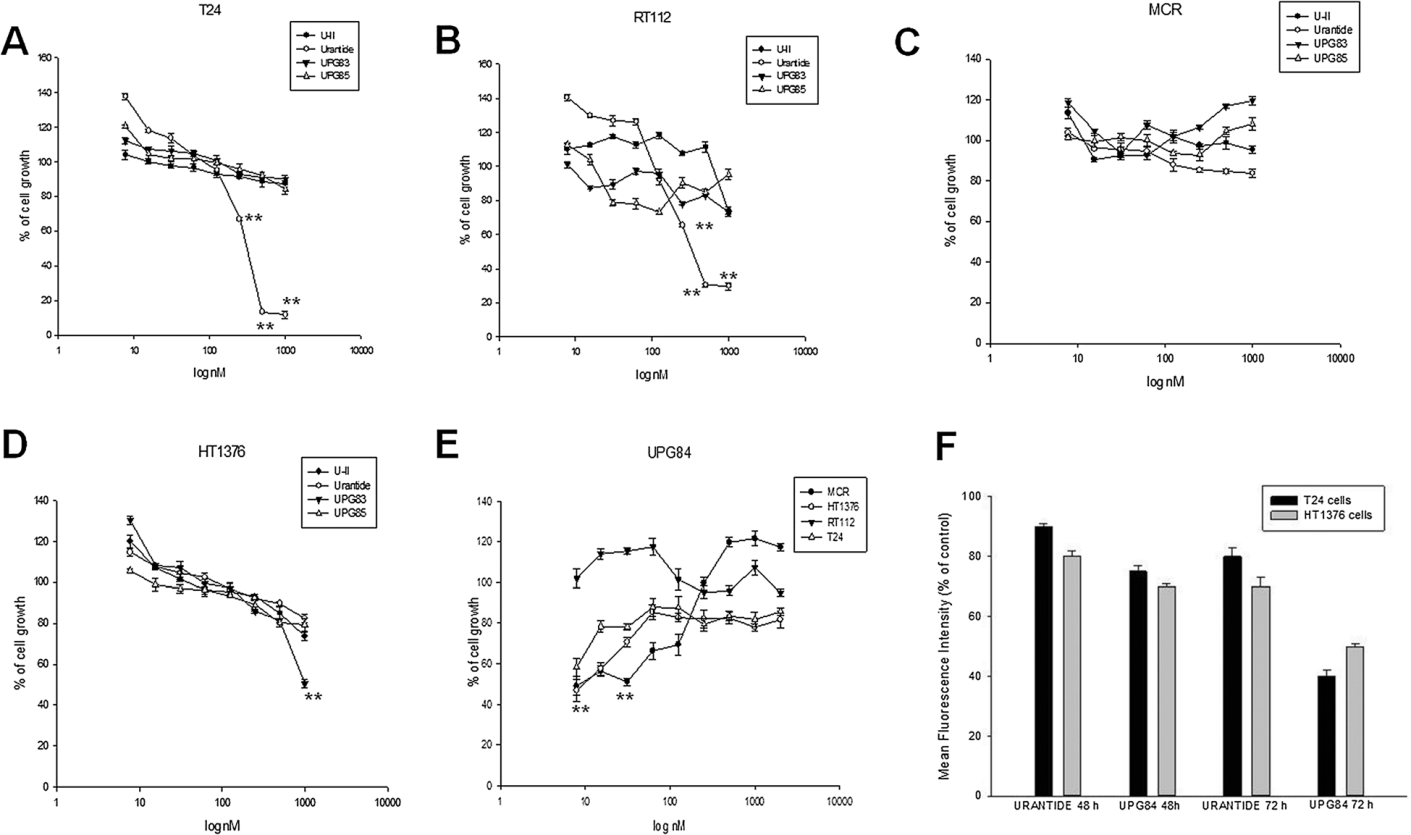
**Effects of agonist (UII), antagonist (Urantide, UPG83 and UPG85) and superagonist (UPG84) on bladder cancer cell growth.** T24 **(A)**, RT112 **(B)** MCR **(C)** and HT1376 **(D)** cells were treated with UII, urantide, UPG83, UPG85 at concentrations ranging from 10 to 1,000 nM, and with UPG84 at concentrations ranging from 10 to 2,000 nM **(E)**, for 72 h at 37°C as described in “Methods” section.% of cell growth was calculated respect untreated cells at 72 h from the beginning of the treatment. Same doses were used for the treatment at 48 h but in these cell lines, the 50% inhibition of cell growth was achieved only at high concentration (T24) or was not reached (RT112). Asterisks indicate the statistical significance of the data (P <0.005). **(F)** HT-1376 (grey) and T24 cells (black) were cultured in the presence or absence of either 10 nM urantide or UPG84 and the expression of UTR was evaluated at FACS at 48 and 72 h as reported in “Methods”. The intensity of UTR expression was represented as% Mean fluorescence intensity (MFI) calculated comparing the MFI of cells incubated with the compounds with those of not treated cells. Each value is the mean of at least three different determinations performed in three different experiments. Bars, SEs. The experiments were performed at least three different times and the results were always similar.

In order to evaluate the involvement of UTR-dependent signaling pathway on the growth of bladder cancer cells, biological effects of human agonists (UII and UPG84), and antagonists (urantide, UPG83 and UPG85), were evaluated on proliferation of human bladder cancer cell lines MCR, RT112, T24 and HT1376 (Figure 6) after 72 h of treatment. UII had no significant effects on the proliferation of all cell lines (Figure 7A-D). The 50% growth inhibitory concentration (IC:50) of urantide was 350 nM in T24 and 375 nM in RT112 while it was not achieved in HT1376 and MCR cells (Table 2). UPG84 induced 45-50% growth inhibition at a concentration close to the supposed Kd of UTR (about 10 nM) in all cell lines with the exception of RT112 cells that were almost insensitive (Figure 7E). Interestingly, UPG84 is a superagonist of UTR (Additional file 1: Table S1) and the addition to cell culture for 72 h could affect UTR expression on cell surface for internalization process triggering. On this light, we have found that treatment of these cells with UPG84 induced a time-dependent down regulation of UTR expression that reached an about 50% and 60% decrease at 72 h from the beginning of the treatment on HT-1376 and T24 cells, respectively (Figure 7F). On the other hand, urantide induced 30 and 20% decrease of UTR expression in HT-1376 and T24 cells, respectively (Figure 7F).

**Table 2:** IC:50s at 72 h of different UTR agonists and antagonists on bladder cancer cell lines

**Cell lines**	**Compounds IC:50 (nM)**				
	**UII**	**Urantide**	**UPG83**	**UPG85**	**UPG84**
**T24**	n.d.	350 ± 8	n.d.	n.d.	7 ± 0,6
**RT112**	n.d.	375 ± 6	n.d.	n.d.	n.d.
**MCR**	n.d.	n.d	n.d.	n.d.	9,8 ± 0,5
**HT1376**	n.d.	n.d.	1000 ± 9	n.d.	7,9 ± 0,4

n.d.: not detectable; IC:50: drug concentration that induces the 50% of cell growth inhibition after 72 h from the beginning of the treatment; UII: urotensin II.

### Effects of Urantide and UTR knock-down on motility and invasion of bladder cancer cells

**Figure 8:**
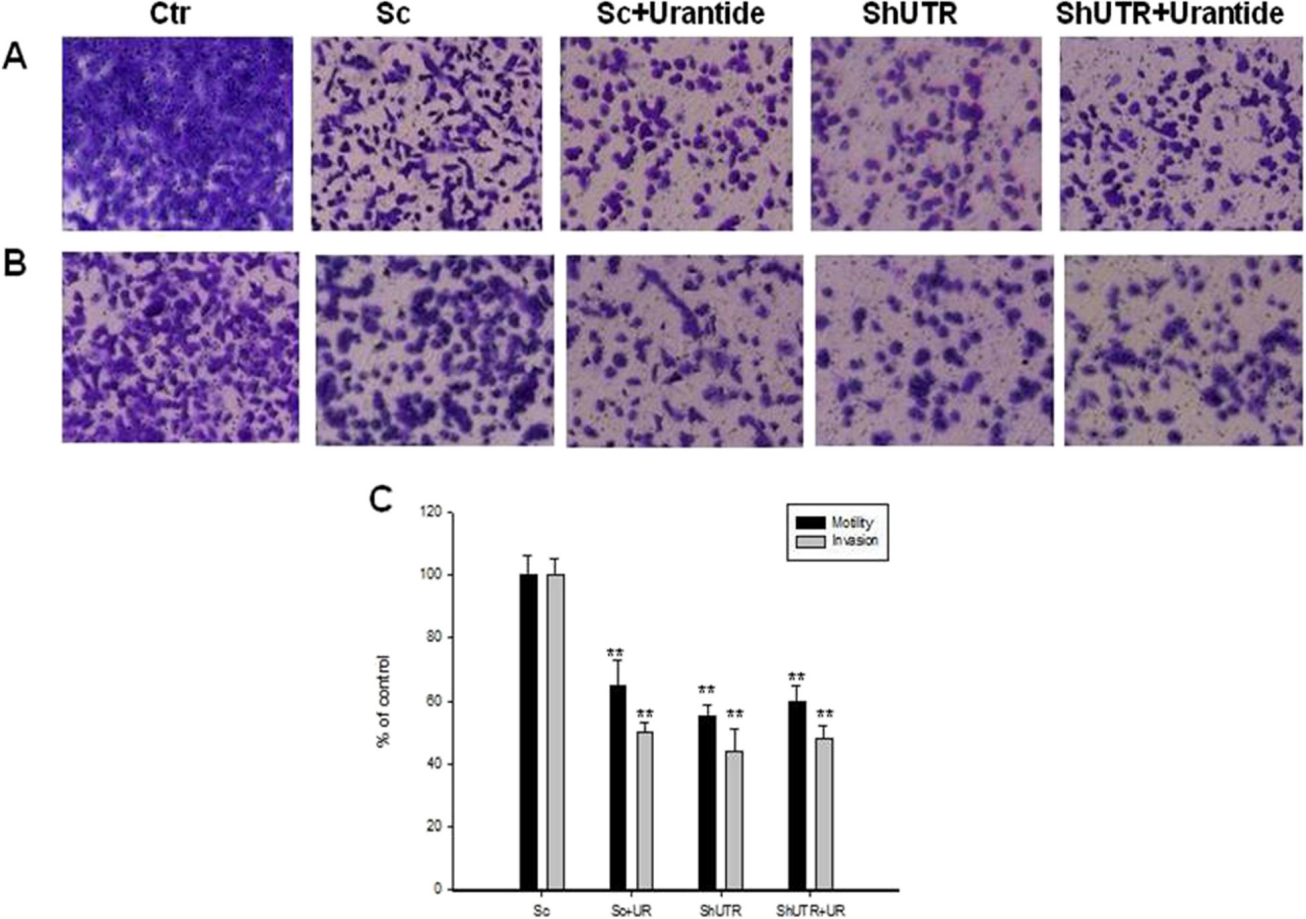
**Effects of down-regulation or block of UTR with either an anti-UTR shRNA or the specific antagonist urantide on cell motility and invasion of T24 cells. A**: T24 parental (CTR) or transiently transfected with a shRNA for UTR (shUTR), were plated in the top chamber of non-coated polyethylene teraphthalate (PET) membranes, treated or not with 100 nM urantide for 48 h and cell motility was evaluated as described in Methods Section. **B**: For in vitro invasion assays, T24 cells were added to a Boyden chamber coated with Matrigel and cell invasion was evaluated as described in Methods Section. The migrating and the invading cells were stained with 0.25% crystal violet for 10 min and photographed under a microscope. **C**: The histogram shows the quantification of the migrating and invading cells measured with a spectrophotometer as OD, and the results are expressed as a percentage as compared to untreated T24 parental cells. The experiments were performed three different times and the results are the mean of the obtained values. Bars, SDs. CTR, untreated T24 cells; Sc, T24 cells transfected with scrambled vector and cultured for 48 h; Sc + UR, T24 cells transfected with scrambled vector and exposed for 48 h to 100 nM urantide; shUTR, T24 cells transfected with shUTR and cultured for 48 h; shUTR + UR, T24 cells transfected with shUTR and exposed to 100 nM urantide for 48 h. Asterisks indicate the statistical significance of the data (P <0.005).

**Figure 9:**
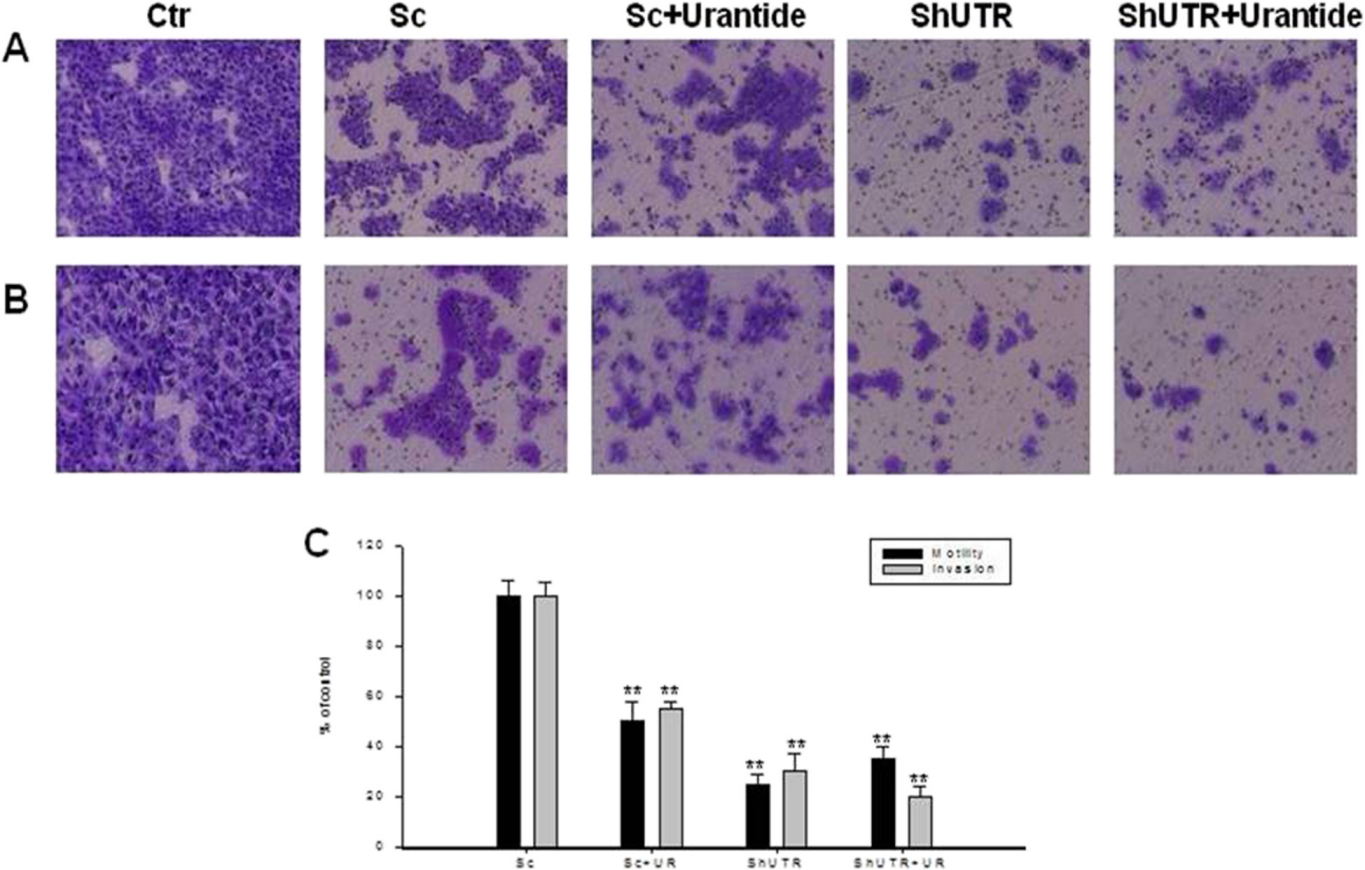
**Effects of down-regulation or block of UTR with either an anti-UTR shRNA or the specific antagonist urantide on cell motility and invasion of RT112 cells. A**: RT112 parental (CTR) or transiently transfected with a shRNA for UTR (shUTR), were plated in the top chamber of non-coated polyethylene teraphthalate (PET) membranes, treated or not with 100 nM urantide for 48 h and cell motility was evaluated as described in Methods Section. **B**: For in vitro invasion assays, RT112 cells were added to a Boyden chamber coated with Matrigel and cell invasion was evaluated as described in Methods Section. The migrating and the invading cells were stained with 0.25% crystal violet for 10 min and photographed under a microscope. **C**: The histogram shows the quantification of the migrating and invading cells measured with a spectrophotometer as OD, and the results are expressed as a percentage as compared to untreated RT112 parental cells. The experiments were performed three different times and the results are the mean of the obtained values. Bars, SDs. CTR, untreated RT112 cells; Sc, RT112 cells transfected with scrambled vector and cultured for 48 h; Sc + UR, RT112 cells transfected with scrambled vector and exposed for 48 h to 100 nM urantide; shUTR, RT112 cells transfected with shUTR and cultured for 48 h; shUTR + UR, RT112 cells transfected with shUTR and exposed to 100 nM urantide for 48 h. Asterisks indicate the statistical significance of the data (P <0.005).

In order to explore the specific contribution of UTR in regulation of motility and invasion of bladder cancer cells, T24 and RT112 cells were treated with urantide (100 nM) for 48 h and/or were transiently transfected with shRNA for UTR to down-regulate UTR protein expression. Cells were seeded in transwell chambers and allowed to migrate and invade in absence or presence of urantide. After 48 h, urantide induced an about 35% and 50% reduction of cell motility and invasion, respectively, in T24 cells if compared to untreated cells. When T24 cells were transfected with shUTR displayed 45% and 56% inhibition of their ability to migrate and invade, respectively (Figure 8A and B, respectively). Downregulation of UTR in T24 treated with urantide did not increase the effect induced by urantide alone (Figure 8A and B, respectively). Similar results were also obtained in RT112 (Figure 9A and B). These data demonstrated that UTR is involved in both motility and invasion of human bladder cancer cells.

## Discussion

NMIBC is characterized by a wide range of neoplastic proliferation with a high propensity to recur and a not negligible tendency to progress to MIBC [21]. Currently, both tumour stage and grade at diagnosis are considered the most important predicting factors of progression in NMIBC [22,23]. Interpretation of histopathologic criteria can also be hampered by significant variability among pathologists [24]. On these bases, molecular markers predictive of progression in NMIBC are urgently needed. Identification of gene expression profile of NMIBC helped to prognostic stratification of patients, in order to recognize high-risk patients in terms of tumor progression [25]. However, although extensive efforts, no promising markers suggested by several groups have yet been able to meet these criteria [26].

UII and its receptor are widely expressed and UII represents a potent endogenous vasoconstrictor with physiological mechanisms similar to other potent mediators [27]. In literature, there are several evidences that associate the altered expression of UTR in many cell lines and tumor tissues [28,29].

In some cases it is described an up-regulation of UTR/UII pathway, in others a down regulation correlated to tumour progression [14,15]. In this light, we have previously demonstrated that UTR expression correlates with good prognosis of prostate cancer and is able to discriminate patients with good from those with bad prognosis and with Gleason grade more than 7 and we have also reported that UTR is likely involved in the regulation of prostate cancer cell motility and invasion [12].

On these bases, we have analysed UTR expression in bTCC samples. In this manuscript, we clearly show that UTR expression is able to discriminate between NMIBC and MIBC and is an independent predictive marker of relapse in NMIBC.

UTR mRNA expression on a selected group of bladder cancers suggested that the regulation of UTR in bladder cancers was dependent upon its mRNA levels.

We also investigated upon UTR biological role in bladder cancer cell lines. We have assessed biological effects of human agonist (UII), synthetic agonist (UPG84) and antagonists (urantide, UPG83 and UPG85) on the proliferation of human bladder cancer cell lines. UII, UPG85 and UPG83 had no significant effects on cell growth while UTR antagonists urantide and superagonist UPG84 inhibited cell growth. Interestingly, the latter was more potent than urantide reaching an about 50% growth inhibition after 72 h of treatment on 3 out of 4 cell lines examined at a concentration near to the supposed Kd of UTR. This effect could be ascribed to UTR downregulation induced by the superagonist likely due to the induction of its internalization and subsequent degradation.

Since UTR seems to be involved in the regulation of intracellular Ca^++^ levels correlated with cell contraction and cytoskeleton changes, we have evaluated effects of shUTR and urantide on both motility and invasion of RT112 and T24 cells [30,31]. Indeed, we found that downregulation of either the function or expression of UTR had significant effects on both motility and invasion of bladder cancer cells and that its down-regulation caused by the specific shRNA induced biological effects similar to those triggered by the addition of antagonist in T24 and was even more potent in RT112.

## Conclusions

We have studied the biological role of UTR in bladder cancer and obtained data suggesting its involvement in the regulation of cell motility and invasion. These data suggest that UII/UTR mediated pathway may play a role in bladder cancer progression. Finally, UTR expression can be an independent predictive factor of progression in NMIBC patients.

## Abbreviations

NMIBC: Non muscle invasive bladder transitional cancer; MIBC: Muscle invasive bladder transitional cancer; UII: Urotensin-II; UTR: Urotensin-II receptor; bTCC: Bladder transitional cell carcinoma; CIS: Carcinoma in situ; VEGF: Vascular endothelial growth factor; DFS: Disease free survival; OS: Overall survival; TMA: Tissue micro-array.

## Competing interest

The authors declare that they have no competing interests.

## Authors’ contributions

RF, MCa, GB and VG were involved in the immunohistochemistry experiments; MCa and MCe were involved in real time PCR experiments made on paraffin embedded tissues; GV was involved in the collection of data and classification of tissue samples; SZ, AL were involved in all cell biology (including transfections and evaluation of cell growth, migration and invasion) and western blotting experiments; SC, GF, SPi, GDL, SPe, MDS were involved in the collection of data regarding the clinical follow-up of the patients; AS was involved in statistical analysis; AMY, EN and PG were involved in the synthesis of peptides used in the study; RF, PG and MC were involved in the experimental design, interpretation of the results and paper writing. All authors read and approved the final manuscript.

## Supplementary Material

Additional file 1: Table S1 Receptor Affinity and Biological Activity of Analogues of Urotensin-II used in this study. Click here for file
