# Supplementary material for: Urotensin II receptor determines prognosis of bladder cancer regulating cell motility/invasion
Source: J Exp Clin Cancer Res. 2014 Jun 3;33(1):48. doi: 10.1186/1756-9966-33-48 (PMC4061920; doi:10.1186/1756-9966-33-48)
Supplement: Additional file 1: Table S1 — Receptor Affinity and Biological Activity of Analogues of Urotensin-II used in this study. [file 1756-9966-33-48-S1.doc]

**Additional file 1: Table S1.** Receptor Affinity and Biological Activity of Analogues of Urotensin-II used in this study:

H-Asp-c[Pen*^a^*-Phe-*Xaa*-*Yaa*-R-Cys]-Val-OH

| Peptide | Xaa | Yaa | R | pK_i_ ^b^ | pD_2_ ^c^ | E_max_^d^ | pK_B_^e^ |
| --- | --- | --- | --- | --- | --- | --- | --- |
| *h*U-II | Trp | Lys | Tyr | 9.10 ±0.08 | 8.310 | 100 | - |
| *h*U-II(4-11) | Trp | Lys | Tyr | 9.60±0.07 | 8.437 | 100 | - |
| Urantide | DTrp | Orn | Tyr | 8.30 ± 0.04 | Inactive | 14±4 | 8.30 |
| UPG83 | DTrp | Orn | (pCN)Phe | 7.92±0.07 | - | 0.0 | 8.148 |
| UPG85 | DTrp | Orn | (pNH_2_)Phe | 7.87±0.01 | - | 0.0 | 7.86 |
| UPG95 | DTrp | Orn | (pNO_2_)Phe | 7.77±0.08 | - | 0.0 | 8.116 |

^a^ Cys in *h*U-II and *h*U-II(4-11); ^b^ pKi: -log Ki; ^c^ pEC_50_: -log EC_50_; ^d^ percent versus hUTII; ^e^ pK_B_ (-log K_B_) values are from experiments in the rat thoracic aorta. Each value in the table is mean ± s.e.m. of at least 4 determinations. * H-Glu-Thr-Pro-Asp-
